# Heart rate variability during slow wave sleep is linked to functional connectivity in the central autonomic network

**DOI:** 10.1093/braincomms/fcad129

**Published:** 2023-05-24

**Authors:** Shawn D X Kong, Christopher J Gordon, Camilla M Hoyos, Rick Wassing, Angela D’Rozario, Loren Mowszowski, Catriona Ireland, Jake R Palmer, Ronald R Grunstein, James M Shine, Andrew C McKinnon, Sharon L Naismith

**Affiliations:** Healthy Brain Ageing Program, Brain and Mind Centre, University of Sydney, Camperdown, NSW 2050, Australia; Charles Perkins Centre, University of Sydney, Camperdown, NSW 2050, Australia; School of Psychology, Faculty of Science, University of Sydney, Camperdown, NSW 2050, Australia; CIRUS, Centre for Sleep and Chronobiology, Woolcock Institute of Medical Research, University of Sydney, Glebe, NSW 2037, Australia; Faculty of Medicine and Health, University of Sydney, Camperdown, NSW 2050, Australia; Healthy Brain Ageing Program, Brain and Mind Centre, University of Sydney, Camperdown, NSW 2050, Australia; Charles Perkins Centre, University of Sydney, Camperdown, NSW 2050, Australia; School of Psychology, Faculty of Science, University of Sydney, Camperdown, NSW 2050, Australia; CIRUS, Centre for Sleep and Chronobiology, Woolcock Institute of Medical Research, University of Sydney, Glebe, NSW 2037, Australia; CIRUS, Centre for Sleep and Chronobiology, Woolcock Institute of Medical Research, University of Sydney, Glebe, NSW 2037, Australia; Healthy Brain Ageing Program, Brain and Mind Centre, University of Sydney, Camperdown, NSW 2050, Australia; School of Psychology, Faculty of Science, University of Sydney, Camperdown, NSW 2050, Australia; CIRUS, Centre for Sleep and Chronobiology, Woolcock Institute of Medical Research, University of Sydney, Glebe, NSW 2037, Australia; Healthy Brain Ageing Program, Brain and Mind Centre, University of Sydney, Camperdown, NSW 2050, Australia; Charles Perkins Centre, University of Sydney, Camperdown, NSW 2050, Australia; School of Psychology, Faculty of Science, University of Sydney, Camperdown, NSW 2050, Australia; Healthy Brain Ageing Program, Brain and Mind Centre, University of Sydney, Camperdown, NSW 2050, Australia; Healthy Brain Ageing Program, Brain and Mind Centre, University of Sydney, Camperdown, NSW 2050, Australia; CIRUS, Centre for Sleep and Chronobiology, Woolcock Institute of Medical Research, University of Sydney, Glebe, NSW 2037, Australia; Faculty of Medicine and Health, University of Sydney, Camperdown, NSW 2050, Australia; Royal Prince Alfred Hospital, University of Sydney, Camperdown, NSW 2050, Australia; Royal Prince Alfred Hospital, University of Sydney, Camperdown, NSW 2050, Australia; Healthy Brain Ageing Program, Brain and Mind Centre, University of Sydney, Camperdown, NSW 2050, Australia; Charles Perkins Centre, University of Sydney, Camperdown, NSW 2050, Australia; School of Psychology, Faculty of Science, University of Sydney, Camperdown, NSW 2050, Australia; Healthy Brain Ageing Program, Brain and Mind Centre, University of Sydney, Camperdown, NSW 2050, Australia; Charles Perkins Centre, University of Sydney, Camperdown, NSW 2050, Australia; School of Psychology, Faculty of Science, University of Sydney, Camperdown, NSW 2050, Australia

**Keywords:** central autonomic network, autonomic function, heart rate variability, sleep

## Abstract

Reduced heart rate variability can be an early sign of autonomic dysfunction in neurodegenerative diseases and may be related to brain dysfunction in the central autonomic network. As yet, such autonomic dysfunction has not been examined during sleep—which is an ideal physiological state to study brain–heart interaction as both the central and peripheral nervous systems behave differently compared to during wakefulness. Therefore, the primary aim of the current study was to examine whether heart rate variability during nocturnal sleep, specifically slow wave (deep) sleep, is associated with central autonomic network functional connectivity in older adults ‘at-risk’ of dementia.

Older adults (*n* = 78; age range = 50–88 years; 64% female) attending a memory clinic for cognitive concerns underwent resting-state functional magnetic resonance imaging and an overnight polysomnography. From these, central autonomic network functional connectivity strength and heart rate variability data during sleep were derived, respectively. High-frequency heart rate variability was extracted to index parasympathetic activity during distinct periods of sleep, including slow wave sleep as well as secondary outcomes of non-rapid eye movement sleep, wake after sleep onset, and rapid eye movement sleep. General linear models were used to examine associations between central autonomic network functional connectivity and high-frequency heart rate variability.

Analyses revealed that increased high-frequency heart rate variability during slow wave sleep was associated with stronger functional connectivity (*F* = 3.98, *P* = 0.022) in two core brain regions within the central autonomic network, the right anterior insular and posterior midcingulate cortex, as well as stronger functional connectivity (*F* = 6.21, *P* = 0.005) between broader central autonomic network brain regions—the right amygdala with three sub-nuclei of the thalamus. There were no significant associations between high-frequency heart rate variability and central autonomic network connectivity during wake after sleep onset or rapid eye movement sleep.

These findings show that in older adults ‘at-risk’ of dementia, parasympathetic regulation during slow wave sleep is uniquely linked to differential functional connectivity within both core and broader central autonomic network brain regions. It is possible that dysfunctional brain–heart interactions manifest primarily during this specific period of sleep known for its role in memory and metabolic clearance. Further studies elucidating the pathophysiology and directionality of this relationship should be conducted to determine if heart rate variability drives neurodegeneration, or if brain degeneration within the central autonomic network promotes aberrant heart rate variability.

## Teaser

Kong *et al.* revealed in older adults ‘at-risk’ of dementia, higher parasympathetic activity during slow wave sleep is associated with increased functional connectivity in both core and broader regions of the central autonomic network. This relationship did not exist for other sleep stages or awake periods at night.

## Graphical Abstract

**Graphical abstract:**
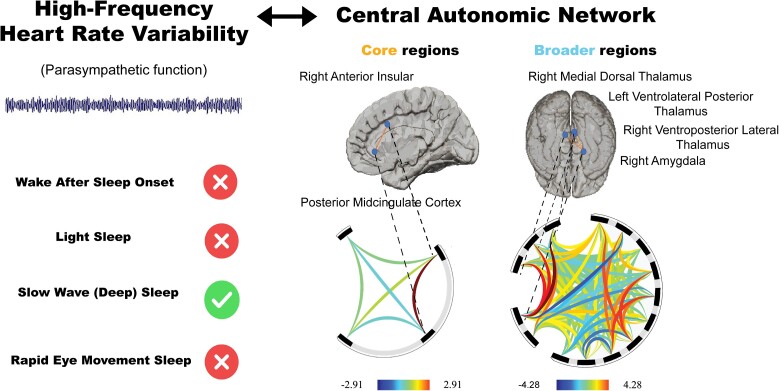

## Introduction

Research in the field of dementia has increasingly focused on studying key neurobiological changes in preclinical or prodromal ‘at-risk’ periods. While older people with subjective cognitive impairment (SCI) are at heightened risk of developing dementia,^1^ those meeting clinical criteria for mild cognitive impairment (MCI; show objective evidence of cognitive deficits on neuropsychological testing),^2^ have a much higher transition rate (i.e. 45%) to dementia within the next five years.^3^ Identifying factors that may be linked to brain and cognitive alterations within these at risk periods is therefore important not only to advance our scientific understanding of the pathophysiology of disease, but to inform the development and delivery of personalized and timely interventions within these selective prevention critical windows.

Despite considerable progress in the field of biomarkers,^4–6^ there remains a relatively poor understanding of changes within the autonomic nervous system (ANS) in dementia or in those at heightened risk of developing dementia. Currently, heart rate variability (HRV) is the most sensitive and non-invasive method to examine cardiovascular autonomic function in humans during rest and sleep.^7^ HRV is a collective term that refers to the variation in the time interval between successive heartbeats with low and high-frequency time-series components, where the high-frequency component (i.e. HF-HRV) reflects vagal activity of the parasympathetic nervous system.^8^ Over the last three decades, there have been only a few studies conducted in various types of dementia, with mixed results regarding the presence of aberrant^9–11^ daytime HRV, as compared to healthy controls.^12–14^ Those that have examined HRV in individuals ‘at-risk’ of dementia have not consistently demonstrated ANS impairment^10,15–18^ but notably these studies only measured short-term daytime HRV (e.g. 5–10 mins) in awake participants.

In particular, there is a paucity of studies measuring HRV during sleep, when the ANS shows differential activation patterns.^19^ Sleep is an ideal state to examine ANS activity as different sleep stages show varying autonomic activation patterns.^20^ From analysis of sleep macro-architecture (i.e. sleep stages), parasympathetic activity predominates during non-rapid eye movement (NREM) sleep^21^ and in particular, during slow wave sleep (SWS).^22^ SWS is characterized by high-amplitude synchronized electroencephalographic (EEG) delta waves, known as slow wave activity (0.5–4 Hz),^23^ aspects of sleep ‘micro-architecture’ which are important in memory consolidation.^24^ In humans, although age-related changes in sleep macro- and micro-architecture exist throughout the entire lifespan, reduced duration and fragmentation of SWS are the most profound sleep alterations in later life.^25^ In addition, SWS changes tend to be exacerbated in late life sleep disorders and neurodegenerative disease. For instance, loss of slow wave activity has been shown to predict prospective increase in cortical beta-amyloid (i.e. pathophysiological biomarker of Alzheimer’s disease) accumulation in cognitively normal older adults.^26^ MCI patients with an amnestic profile (i.e. amnestic MCI) are at most pronounced risk of developing Alzheimer’s disease. Prior work has shown that such individuals have reduced SWS time and delta power during sleep compared to age-matched healthy adults.^27^ Our group further demonstrated that parasympathetic activity (indexed by HF-HRV) during SWS is reduced in older adults at risk for dementia, and this effect was especially pronounced in those with the amnestic MCI subtype.^28^ Therefore, parasympathetic activity during SWS might be an early peripheral pathophysiological biomarker of neurodegeneration that warrants further examination in relation to central nervous system (CNS) integrity.

Our current understanding of how ANS activity interacts with the CNS generally, and brain degeneration and cognitive decline specifically is nascent. An intricate network of brain regions collectively known as the central autonomic network (CAN)^29–31^ governs the top–down regulation of the ANS. The CAN is comprised of a series of brainstem nuclei,^32–34^ connected to cortico-limbic structures involved in behavioral, cognitive and sleep regulation.^34–36^ Previous research has primarily employed task-based functional magnetic resonance imaging (fMRI) to investigate the relationship between daytime HRV and the CAN, whereby concurrent changes in neural activation and autonomic functioning are induced as a result of experimental manipulation. In a comprehensive meta-analytic review of 43 task-based fMRI studies, four core regions of the CAN (i.e. the left amygdala, right anterior insula, left posterior insula, and the posterior midcingulate cortex) were identified to be consistently involved in autonomic modulation during all task modalities,^37^ suggesting activation in the CAN contributes to autonomic responses to cognitive, emotional and somatosensory tasks.

In recent years, an increasing number of studies have employed resting-state fMRI (rsfMRI) instead of task-based fMRI to study CAN integrity. In addition to the ability to identify cohesive functional networks that demonstrate connectivity in the absence of performing a task,^38^ rsfMRI is ideal for patient groups that may be unable to perform certain cognitive tasks (e.g. due to MCI or dementia).^34^ However, rsfMRI studies investigating the CAN while incorporating HRV remain relatively sparse in the extant literature.^39–41^ So far, a novel discovery enabled by the use of rsfMRI (i.e. not been apparent from prior task-based fMRI studies of the CAN^37^) has revealed that there is increased functional connectivity between the thalamus and other brain regions during states of elevated HF-HRV.^40,41^ Interestingly, specific thalamic nuclei (ventrolateral posterior, ventroposterior lateral, and medial dorsal) demonstrate temporally synchronous relationships with HF-HRV fluctuations, suggesting that the thalamus is integral to the regulation of autonomic outflow.^41^ Importantly, the brain regions implicated in CAN activities in the above study overlapped in whole or in part with three of the four regions (i.e. right anterior insula, left posterior insula, and midcingulate cortices) previously identified under task-based conditions by Beissner *et al*.,^37^ suggesting that these CAN areas are implicated in ANS regulation regardless of task or rest.

From the literature to date, there remains a clear gap in our understanding of how HRV during sleep is associated with brain functional connectivity in older adults ‘at-risk’ of dementia. This is important to understand, because if lower levels of HF-HRV during sleep are etiologically linked to reductions in the cohesion of functional connectivity between brain regions, it may provide a window of opportunity for early heart-brain interventions. Therefore, the primary aim of the study was to examine in older people ‘at-risk’ of dementia whether HRV during SWS is associated with the strength of functional connectivity within: (i) the four core CAN regions implicated ANS regulation;^37^ and (ii) across the broader set of brain regions recently implicated in the CAN’s regulation of the ANS during the resting-state.^41^

## Materials and methods

### Participants

Older adults with cognitive concerns were recruited from the Healthy Brain Ageing Clinic, a specialist memory and cognition research clinic at the Brain and Mind Centre, University of Sydney, Australia. Specific inclusion criteria were: age ≥50 years, subjective cognitive decline, mini-mental state examination (MMSE) ≥ 20; and referral from a general practitioner or medical specialist. Exclusion criteria for the clinic were: stroke, transient ischemic attack, head injury with loss of consciousness >30 min; diagnosed or detected neurological conditions; other medical conditions known to affect cognition; intellectual disability, history of substance abuse; major psychiatric disorders (with the exception of depression/anxiety); and insufficient English proficiency for standardized testing. For this particular study, additional exclusion criteria were: diagnosed cardiovascular conditions (i.e. severe ischemic heart disease, unstable tachycardia, severe valvular heart disease, non-sinus rhythm including atrial fibrillation and other arrhythmias, paced rhythms); regular use of beta-blockers; and/or centrally-active antihypertensive drugs. This study was approved by the University of Sydney Human Research Ethics Committee (Protocol number: 2012/1873). All participants provided written informed consent prior to study participation.

### Procedure and measures

Participants underwent standardized medical, mood and neuropsychological assessments followed by an overnight polysomnography (PSG) sleep study and a magnetic resonance imaging (MRI) scan.

#### Medical assessment

A neurologist or geriatrician conducted a physical examination and recorded medical history, including alcohol consumption per week, history of smoking, and current medication (including antidepressant medication use), using a semi-structured interview. Illness burden was assessed using the Cumulative Illness Rating Scale Geriatric Version.^42^ The MMSE was administered for reporting and screening purposes. Additionally, body mass index was derived from anthropometric measures.

#### Mood assessment

History of psychiatric illness was determined using structured clinical interview questions from the Mini International Neuropsychiatric Interview.^43^ Additionally, participants self-reported their current depressive symptoms using the 15-item geriatric depression scale (GDS).^44^

#### Neuropsychological assessment and ‘at-risk’ classifications

A Clinical Neuropsychologist conducted a standardized neuropsychological assessment using tests sensitive to ageing and neurodegeneration, as published previously.^45^ Predicted intellectual ability was gathered using the Wechsler Test of Adult Reading (WTAR).^46^ Following the assessment, a consensus meeting was held and included a geriatrician and two neuropsychologists. On the basis of clinical history and medical, mood, and neuropsychological assessment, individuals were classified as having either (i) SCI (subjective complaints) or (ii) MCI, using the Winblad clinical criteria.^47^

### Polysomnography and HRV data acquisition and analysis

As detailed previously,^28^ the overnight PSG included 6-channel electroencephalography in accordance with the 10–20 configuration and electrocardiography (ECG) in accordance with the standard lead II configuration. Signals were digitized at 512 Hz. The PSG recording was scored by an experienced sleep technician. Sleep macro-architecture measures [e.g. total sleep time, sleep efficiency, wake after sleep onset (WASO), N1, N2, N3, and REM sleep, where N3 was defined as SWS] and sleep events (e.g. EEG arousals, apnea hypopnea index) were visually scored according to standard criteria.^48^

As previously described,^28^ ECG signals were processed with PRANA software v.12.06.20 (PhiTools, Strasbourg, France) to derive HRV data. The ECG signal from lights-off to lights-on was filtered using a band-pass filter of between 0.3 and 70 Hz. The ECG signal was subjected to a validated QRS-complex detection algorithm (PhiTools, Strasbourg, France), from which the instantaneous inter-beat interval timeseries were calculated. In addition, the data were inspected and manually corrected by SDK to ensure accurate inter-beat interval identification. Recordings with more than 20% artefacts or repeated cardiac dysrhythmias were excluded. After that, fast Fourier transform analysis was applied to the inter-beat interval timeseries to calculate frequency-domain HRV indices, whereby HF-HRV was employed to index parasympathetic activity.^49^ To correct for the interindividual differences in total power, we followed standard recommendation to report HF-HRV in normalized units, calculated by high frequency power/(total power - very low frequency power)*100.^50^

### Neuroimaging analysis

#### Image acquisition

T1-weighted structural MRI scans and rsfMRI scans were obtained using a GE MR750 3-Tesla MRI (General Electric, Milwaukee, USA) at the Brain and Mind Centre, Sydney. The scan was performed within four weeks of the overnight PSG study. The structural data were acquired using used an 8-channel head coil, and a 3D-T1-weighted BRAVO Spoiled Gradient-Recalled sequence with 196 sagittal slices (repetition time = 7.2 ms; echo time = 2.8 ms; flip angle = 12°; matrix 256 × 256; 0.9 mm isotropic voxels). Resting-state fMRI data were acquired using a T2*-weighted echo planer imaging sequence (39 axial slices covering the whole brain; TR = 3000 ms, TE = 36 ms, flip angle = 90°, matrix: 64 × 64, in-plane voxel size = 3.75 mm × 3.75 mm × 3 mm). The first five volume acquisitions of the resting-state echo planer imaging data were discarded to eliminate spurious T2-equilibration effects, before a further 140 echo planer imaging-volumes were acquired in a single run, where each participant lay supine in the scanner with their eyes closed and was instructed to allow their mind to wander.

#### Resting-state fMRI pre-processing

Image processing and analysis was performed using the functional connectivity toolbox (CONN v19.c; http://www.nitrc.org/projects/conn) in MATLAB (MATLAB and Statistics Toolbox Release 2015, The Mathworks Inc., Natick, Massachusetts, US). Scans underwent pre-processing utilizing a standardized Montreal Neurological Institute (MNI)-space direct normalization pipeline—see Nieto-Castanon^51^ for complete details. In short, the echo planer imaging volumes were co-registered and resampled to a reference volume (the first scan) using b-spline interpolation. From this registration, rigid head movement timeseries were calculated (6 degree of freedom) and added as a first-level covariate for correction of minor head movements (see aCompCor below). Slice-timing correction using sinc-interpolation to match the mid-TR time was then performed. In addition, outlier scans were identified and defined as volumes with frame-wise displacement greater than 0.5 mm or signal intensity changes greater than three standard deviations (SDs). Functional and structural data were subsequently normalised into standard MNI space and segmented into grey matter, white matter, and cerebrospinal fluid tissue classes using the SPM12 unified segmentation and normalization procedure, which includes estimating the best non-linear spatial transformation for the data.^52^ This procedure was applied separately to the functional data, using the mean blood-oxygen-level-dependent signal as the reference image, and to the structural data, using the raw T1-weighted volume as the reference image. Data were resampled to 2 mm isotropic voxels for functional data and 1 mm isotropic voxels for structural data, using 4th order spline interpolation.

#### Denoising, quality assurance, and time course correlations

After pre-processing, a standardized denoising pipeline was implemented which combined two general steps. In short, a noise correction procedure (aCompCor) was applied to the blood-oxygen-level-dependent timeseries to regress out noise and motion artefacts. For this procedure, noise regressors were obtained from the white matter and cerebrospinal fluid timeseries, as well as the first-level covariates previously defined (12 components from the estimated subject-motion parameters, derived from three translation and three rotation parameters and their first-order derivatives, and the outlier volumes to be ‘scrubbed’). A temporal band pass filter was applied to the blood-oxygen-level-dependent signal (0.009–0.08 Hz) after confound regression to minimize the influence of physiological, head-motion and other noise sources. This was performed after regression to avoid any frequency mismatch in the nuisance regression procedure.^53^ Images were then visually inspected for quality.

### Statistical analysis

#### Resting-state fMRI functional connectivity analysis

Regions of interest (ROIs) were chosen based on the two seminal works in this area—the pooled task-based fMRI meta-analysis by Beissner *et al*.,^37^ as well as the Valenza *et al.*^41^ simultaneous HRV-rsfMRI study. An ROI-to-ROI functional connectivity analysis was conducted using 8 mm spherical ROIs. Placement of ROIs was based on their purported relevance to autonomic function and previously published MNI-coordinates as defined by the seminal works previously reviewed. The 15 ROIs comprised the bilateral posterior (pMCC) and right anterior midcingulate cortex (aMCC); the bilateral anterior (aINS), right dorsomedial (dmINS), and left posterior (pINS) insular cortices, right paracentral lobule, left superior frontal gyrus, and several thalamic regions: bilateral medial dorsal (mdTH), left ventrolateral posterior (vlpTH), and right ventroposterior lateral (vplTH) thalamic nuclei. Additionally, we used 8 mm spherical ROIs for the left (MNI: -23, -5, -18) and right (MNI: 23, -4, -18) amygdala, derived from the CONN atlas. The CONN atlas was chosen to define amygdalae coordinates as these were not consistent across the Beissner *et al.*^37^ and Valenza *et al.*^41^ papers, and involved some overlap with surrounding anatomical regions. Using this approach, we were able to better isolate the signal from the amygdalae only and create the relevant ROIs for the current study.

The mean blood-oxygen-level-dependent signal time courses were then extracted from each ROI. Time courses for each ROI were then correlated with the time courses for all other ROIs, producing Pearson’s *r*-values that were subsequently converted into *z*-scores using a Fisher’s *r*-to-*z* transformation.

### Statistical analysis

Utilizing the CONN toolbox, whole-sample statistical analyses were conducted to evaluate associations in our ‘at-risk’ older adult sample between HF-HRV during various sleep stages (i.e. HF-HRV_SWS,_ HF-HRV_WASO_, HF-HRV_NREM_, and HF-HRV_REM_) and functional connectivity within the CAN. General linear models were implemented for these analyses, controlling for the potential confounding effects of age, sex, and antidepressant usage. Specifically, cluster-level inferences for functional network connectivity were used;^54^ where cluster threshold: *P* < 0.05 cluster-level *P*-FDR corrected (MVPA omnibus test); and connection threshold: *P* < 0.05 uncorrected. Unpaired *t*-tests were carried out on each resting-state functional connection to determine whether there were any HF-HRV and connectivity associations. The significance threshold was determined the false discovery rate correction method (FDR) with α set to 0.05.^55^*P*-values reported throughout this study with respect to functional connectivity analyses are the FDR-corrected values, and beta values reported are unstandardized. Results are unlikely to be driven by between-group differences in head movement and global signal (both *P* > 0.36).

### Data availability

The data underlying this article cannot be shared publicly to protect the privacy of individuals that participated in the study. The study ethics also prohibits public sharing of data. De-identified data will be shared on a reasonable request to the corresponding author.

## Results

### Demographics

As shown in Table 1, 78 older adults (mean age = 67.1, SD = 9.4, range = 50–88 years; 64% female) were recruited. For descriptive purposes, sleep macro-architecture features derived from the PSG were reported in Table 2, whereas heart rate and HF-HRV indices during different sleep periods were reported in Table 3. On average, participants reported 13–15 years of education, with a mean IQ in the average range (mean WTAR = 106.4), and normal levels of subjective depressive symptoms (i.e. mean GDS total score < 4), and objective sleep efficiency of 78% derived from PSG. Eight patients met criteria for a current major depressive episode, 13 participants were taking antidepressant medications (e.g. selective serotonin reuptake inhibitors or serotonin–noradrenaline reuptake inhibitors) and two participants were taking benzodiazepines regularly (i.e. 2–3 times per week). There were 25 participants with SCI and 53 meeting clinical criteria for MCI.

**Table 1:** Demographic and clinical characteristics of older adults ‘at-risk’ of dementia^a^

	M (SD)^a^
N	78
Age, years	67.10 (9.4)
Sex, female (%)	50.00 (64.1)
Education, years	14.04 (2.7)
Body Mass Index	26.94 (4.8)
Alcohol consumption, drinks per week	5.22 (7.5)
History of smoking, (%)	36.00 (46.2)
Cumulative Illness Rating Scale-Geriatric	4.48 (3.5)
Geriatric Depression Scale-15	3.49 (3.2)
Lifetime depression history, (%)	35.00 (44.9)
Currently depressed, (%)	8.00 (10.3)
Current anti-depressant use, (%)	13.00 (16.7)
Benzodiazepine use, (%)	2.00 (2.6)
Mini-Mental State Examination,/30	28.45 (2.9)
Wechsler Test of Adult Reading, Premorbid IQ	106.40 (10.0)
Mild Cognitive Impairment, (%)	53.00 (67.9)

a M, mean; SD, standard deviation. All data are reported as M (SD) unless otherwise stated.

**Table 2:** Sleep and breathing indices of older adults ‘at-risk’ of dementia (M, SD)^a^

	M (SD)^a^
Total time in bed (min)	431.15 (47.6)
Total sleep time (min)	341.29 (64.5)
Wake after sleep onset (min)	76.77 (54.9)
Stage 1 (N1) sleep time (min)	31.77 (27.3)
percentages (%)	9.50 (7.8)
Stage 2 (N2) sleep time (min)	170.86 (50.1)
percentages (%)	51.30 (11.5)
Slow wave sleep (N3) sleep time (min)	79.93 (39.2)
percentages (%)	23.00 (10.1)
Non-rapid eye movement sleep time (min)	281.79 (55.5)
percentages (%)	83.80 (10.5)
Rapid eye movement sleep time (min)	59.50 (37.2)
percentages (%)	16.20 (7.3)
Sleep efficiency (%)	77.69 (13.9)
Sleep-onset latency (min)	21.81 (29.7)
Rapid eye movement latency (min)	137.83 (84.8)
Arousals, events per hour	22.08 (15.7)
Awakenings, events per hour	27.49 (18.8)
Apnea Hypopnea Index, events per hour	19.97 (19.2)

a M, mean; SD, standard deviation. All data are reported as M (SD) unless otherwise stated.

**Table 3:** Heart rate and high-frequency heart rate variability (HF-HRV) indices of older adults ‘at-risk’ of dementia (M, SD)^a^

	M (SD)^a^
Wake after sleep onset HF-HRV	23.41 (10.0)
Heart Rate	64.25 (8.4)
Stage 1 (N1) sleep time (min)	25.50 (10.9)
Heart Rate	62.51 (8.2)
Stage 2 (N1) sleep time (min)	28.74 (10.0)
Heart Rate	61.27 (8.1)
Slow wave sleep (N3) HF-HRV	36.08 (13.1)
Heart Rate	61.85 (8.2)
Non-rapid eye movement HF-HRV	30.62 (10.2)
Heart Rate	61.47 (8.1)
Rapid eye movement HF-HRV	24.03 (11.0)
Heart Rate	62.68 (8.7)

a M, mean; SD, standard deviation. All data are reported as M (SD) unless otherwise stated.

### Resting-State connectivity analysis

#### Functional connectivity patterns in the core CAN regions

**Figure 1:**
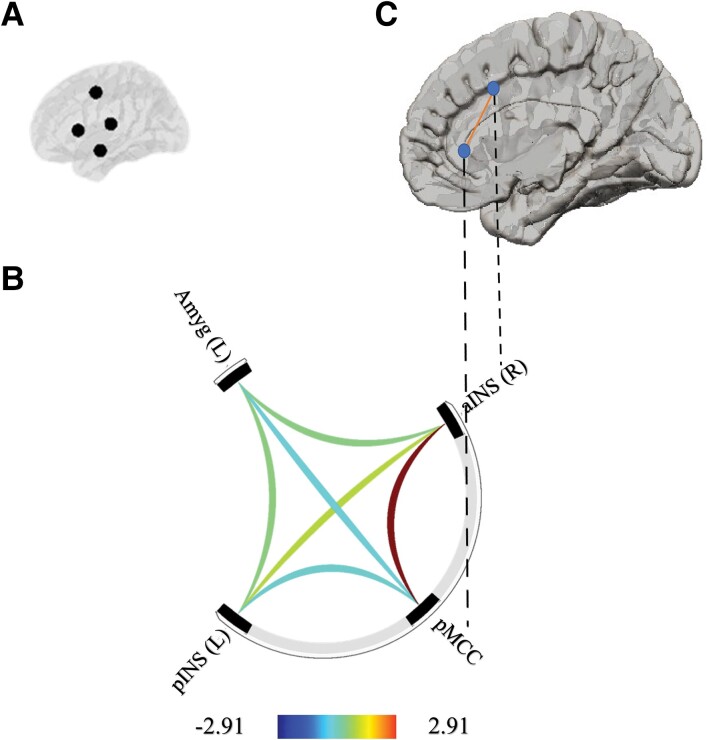
**Functional connectivity patterns in the core brain regions of the central autonomic network (CAN) in older adults ‘at-risk’ of dementia.** Figure (**A**) shows the four core brain regions of the CAN. Figure (**B**) shows the connectome ring of the core CAN functional connectivity where blue indicates decreased functional connectivity and yellow-red indicates increased functional connectivity. This analysis reveals that stronger functional connectivity between the right anterior insula and the posterior midcingulate cortex is most strongly associated with higher HF-HRV during slow wave sleep (HF-HRV_SWS_). Figure (**C**) displays the anatomical regions of interests involved in this finding. aINS R, right anterior insula; pMCC, posterior midcingulate cortex; pINS L, left posterior insula; Amyg L, left amygdala.

A significant association between HF-HRV_SWS_ and functional connectivity strength was found in the four core CAN regions (*F* = 3.98, *P* = 0.022 [FDR-corrected], *P* = 0.011 [uncorrected]). Specifically (Fig. 1), increasing levels of HF-HRV_SWS_ were associated with stronger functional connectivity between the right anterior insula and the posterior midcingulate cortex (beta = 0.00, *t*(73) = −2.91, *P* = 0.014 [FDR-corrected]; *P* = 0.005 [uncorrected]).

There were no significant associations between HF-HRV_WASO_, HF-HRV_NREM_, and HF-HRV_REM_ and functional connectivity strength and the four core CAN regions (i.e. left amygdala, right anterior insula, left posterior insula, posterior midcingulate cortex) (*F*-values all <1.66, *P*-values all >0.317 [FDR-corrected] and >0.186 [uncorrected]).

#### Functional connectivity patterns in the broader CAN regions

Analyses were expanded to include the broader CAN regions (i.e. bilateral anterior insula, left anterior midcingulate cortex, right dorsomedial insula, left posterior insula, right anterior midcingulate cortex, bilateral posterior midcingulate cortex, left ventrolateral posterior thalamus, right ventroposterior lateral thalamus, bilateral medial dorsal thalamus, right paracentral lobule, left superior frontal gyrus) as well as the right amygdala.

**Figure 2:**
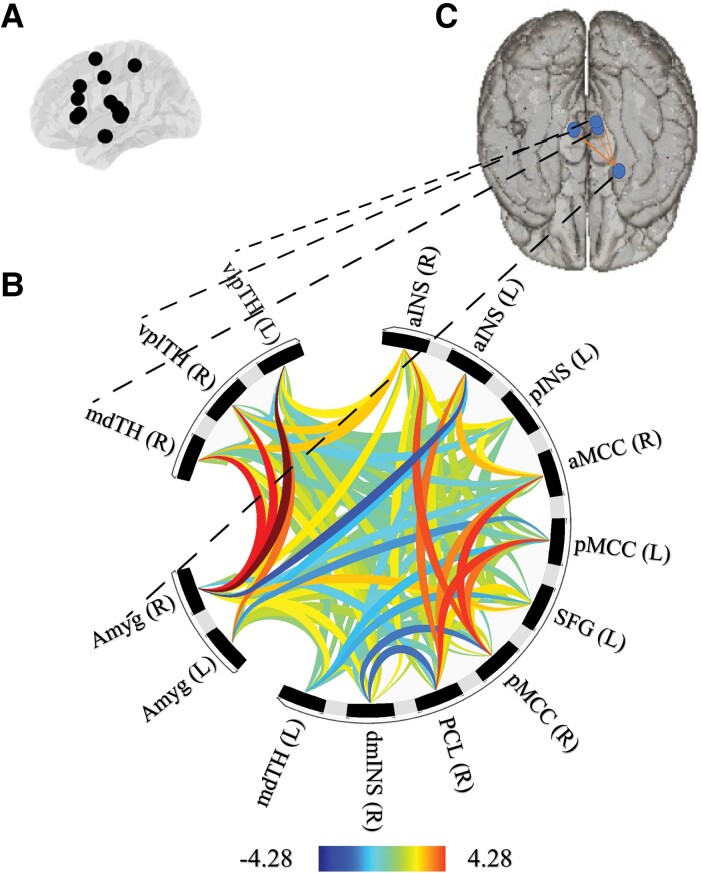
**Functional connectivity patterns in the broader brain regions of the central autonomic network (CAN) in older adults ‘at-risk’ of dementia.** Figure (**A**) shows the fifteen broader brain regions of the CAN (including the core regions). Figure (**B**) shows the connectome ring of the broader CAN functional connectivity where blue indicates decreased functional connectivity and yellow-red indicates increased functional connectivity. This analysis reveals that stronger functional connectivity between the right amygdala (Amyg R) and three sub-nuclei of the thalamus (mdTh R, vplTh R, and vlpTh L) is most strongly associated with higher HF-HRV during slow wave sleep (HF-HRV_SWS_). Figure (**C**) displays the anatomical regions of interests involved in this finding. aINS R, right anterior insula; aINS L, left anterior insula; pINS L, left posterior insula; aMCC R, right anterior midcingulate cortex; pMCC L, left posterior midcingulate cortex; SFG L, left superior frontal gyrus; pMCC R, right posterior midcingulate cortex; PCL R, right paracentral lobule; dmINS R, right dorsomedial insula; mdTH L, left medial dorsal thalamus; Amyg L, left amygdala; Amyg R, right amygdala; mdTH R, right medial dorsal thalamus; vplTH R, right ventroposterior lateral thalamus; vlpTH L, left ventrolateral posterior thalamus.

For HF-HRV_SWS_ specifically, as shown in Fig. 2, there were significant associations between levels of HF-HRV_SWS_ and functional connectivity strength within the broader CAN regions (*F* = 6.21, *P* = 0.005 [FDR-corrected], *P* < 0.001 [uncorrected]). Specifically, higher HF-HRV_SWS_ was associated with stronger functional connectivity between the right amygdala and three specific regions of the thalamus: left ventrolateral posterior thalamus (beta = -0.01, *t*(73) = -4.28, *P* < 0.001 [FDR-corrected], *P* < 0.001 [uncorrected]); right ventroposterior lateral thalamus (beta = -0.00, *t*(73) = -3.30, *P* = 0.010 [FDR-corrected], *P* = 0.002 [uncorrected]); and the right medial dorsal thalamus (beta = -0.01, *t*(73) = -3.19, *P* = 0.010 [FDR-corrected], *P* = 0.002 [uncorrected]). All other connections were non-significant (*F*-values all <1.10; *P*-values all > 0.719 [FDR-corrected], and > 0.360 [uncorrected]).

There was a significant association between levels of HF-HRV_NREM_ and functional connectivity strength within the broader CAN regions (*F* = 4.43, *P* = 0.039 [FDR-corrected], *P* = 0.007 [uncorrected]). Specifically, higher HF-HRV_NREM_ was associated with stronger functional connectivity between the right amygdala and the left ventrolateral posterior thalamus (beta = -0.01, *t*(73) = -3.60, *P* = 0.008 [FDR-corrected], *P* < 0.001 [uncorrected]). All other functional connections in the broader CAN were non-significant for HF-HRV (*F*-values all <3.01; *P*-values all > 0.109 [FDR-corrected], and > 0.0362 [uncorrected]).

There were also no significant associations between levels of HF-HRV_WASO_ or HF-HRV_REM_ and functional connectivity strength (*F*-values all <1.59, *P*-values all > 0.695 [FDR-corrected], and > 0.202 [uncorrected]).

## Discussion

This study sought to examine the relationship between HF-HRV during sleep and CAN resting-state functional connectivity in older people ‘at-risk’ of dementia. Our results demonstrate that higher HF-HRV_SWS_ is linked to increased functional connectivity within both core and broader regions of the CAN. Crucially, these findings were attenuated for HF-HRV_NREM_ and notably absent for HF-HRV_REM_ and HF-HRV_WASO_. These novel findings show that in older adults at elevated risk of dementia, parasympathetic regulation during SWS is strongly associated with disrupted functional connectivity in the CAN.

Interestingly, our findings reveal that during SWS, higher HF-HRV is linked to the *strength of coupling* in the core CAN regions of the bilateral posterior midcingulate and the right anterior insula. These results align with prior CAN studies during task-based,^37^ and rsfMRI,^41^ which showed that the core regions of the CAN—the left amygdala, right anterior insula, left posterior insula, and posterior midcingulate cortex are involved in the autonomic modulation of cognition.^37^ The anterior insula primarily integrates affective and cognitive signals associated with interoceptive abilities such as timing of the heartbeat,^56^ whereas the posterior portion of the cingulate cortex has been consistently shown to be linked to parasympathetic functioning.^29^ Both cortical brain regions have been shown to be implicated in early neurodegenerative processes that contribute to later cognitive decline.^57,58^ This is particularly true for the right insula^59,60^ and posterior cingulate in Alzheimer’s disease with reduced glucose metabolism and increased beta-amyloid accumulation seen using positron emission tomography imaging.^61‐63^ Overall, while we cannot infer causality from this study, our findings are aligned with work suggesting that connectivity within the CAN, and specifically the insula and cingulate cortex, may be important for parasympathetic regulation. That is, connectivity strength may exert ‘top–down’ effects on the brainstem to modulate parasympathetic outflow, which is at its peak during SWS.^19^ Notably, this process would be less likely to be detected during wakefulness where parasympathetic activities are in balance with sympathetic activities, and therefore examination of HF-HRV and brain function may be optimally studied during sleep.

It is important to note that both the cingulate cortex and anterior insula are key nodes of other brain networks. These functionally connected regions are also anatomically close to each other and share similar functions.^64,65^ In fact, the CAN has been shown to play pivotal roles in brain networks associated with emotional, sensory, and cognitive pathways.^34^ It has been posited that the CAN may not be an independent static network, but rather a set of brain regions ‘borrowed’ from other central networks to modulate adaptive psychological or physiological reactions.^41^ For example, the insula is a key hub of the salience network, with other nodes including the amygdala, thalamus, and brainstem. The salience network is believed to be a neural system that is crucial for perceiving and responding to homeostatic demands.^66^ Interactions between the salience network, particularly the right anterior insula, and the default-mode network, a network preferentially but not exclusively active during the resting-state, are believed to be important for cognitive control.^67^ Furthermore, the posterior cingulate cortex is a crucial node of both the CAN^29^ and the default mode network,^68,69^ and one of the early and core regions involved in Alzheimer’s disease pathology. These findings are also aligned with the revised neurovisceral integration model, where an eight-level hierarchy of nervous system structures exist, with each level differentially contributing to vagal control.^70^ New types of information are integrated at each level, with each ascending level more capable of flexibly modifying vagal tone and more engaged with sophisticated self-regulatory functions such as cognitive processing. Given this framework, our findings linking HF-HRV_SWS_ to the coupling of the insula (level 6) and the cingulate cortex (level 7) support the notion that parasympathetic regulation during SWS may be crucial for the integration of cortical systems that are instrumental to higher-order processes such as cognition.

To reach deep sleep (i.e. SWS), there is an approximate 15% reduction in cardiovascular output.^71,72^ As compared to wakefulness, HF-HRV increases significantly during SWS,^73^ accompanied by a decrease in brain activity,^74^ predominantly in subcortical (e.g. brainstem, thalamus, basal ganglia, basal forebrain) and cortical (e.g. prefrontal cortex, anterior cingulate cortex, and precuneus) regions.^75^ Therefore, our finding that stronger amygdala-thalamic functional connectivity is linked to more intact parasympathetic regulation, may be indicative of the unique brain–heart interactions during SWS. For the last few decades, our understanding of the brain areas responsible for sleep generation have extended from the anterior hypothalamus^76,77^ to the posterior portion of hypothalamus,^78,79^ basal ganglia,^80^ and several brainstem nuclei (e.g. medulla;^81^ and periaqueductal grey^82^), indicating that the brain network regulating sleep is functionally and anatomically widely distributed. While previous studies have identified both the thalamus and amygdala to be active during REM sleep,^83^ recent findings reveal the thalamic-amygdala pathway to also be crucial for the promotion of NREM sleep.^84^ In rodents, both the posterior portion of the thalamus and central nucleus of the amygdala expressed neurotensin which promoted NREM sleep. The neurotensin peptide is processed naturally during NREM sleep^85,86^ and knockout of the neurotensin receptor 1 resulted in more wakefulness and less NREM rebound sleep in mice.^87^ Further, the neurotensin peptide is also associated with autonomic regulation such as blood pressure and thermoregulation,^88,89^ which could also be instrumental to the coordination of NREM sleep generation.

Our findings that HF-HRV_SWS_ shows differential associations with functional connectivity strength at both the cortical (right anterior insula and the posterior midcingulate cortex) and subcortical (right amygdala with three specific sub-regions of the thalamus) levels are of particular importance. Given only one of the four significant findings from the HF-HRV_SWS_ analyses remained when examining NREM sleep overall, it is plausible that certain events occur during SWS that are important for maintaining brain network integrity to regulate parasympathetic function. One likely candidate might be the glymphatic system which is primarily responsible for metabolic waste clearance in the brain by facilitating the solute exchange between cerebrospinal fluid and the interstitial fluid.^90^ Importantly, this process is more effective at clearing beta-amyloid during sleep than wakefulness,^91^ and in animal studies, glymphatic clearance has been shown to be optimal during SWS.^92^ A recent study using simultaneous fMRI-EEG during sleep, also showed that cerebrospinal ‘pulsation’ during sleep^93^ might either recruit an autonomic or neural pathway (i.e. generation of slow electrocortical activity), although the exact nature of interactions between the pathways is still unclear. As such, autonomic function could be one of the key drivers of glymphatic clearance.

On the other hand, aging-related reduction in the amount of SWS is primarily attributed to the decrease in the amplitude of delta wave activity, and not necessarily weakened slow frequency activity.^23^ The incidence of low frequency neuronal oscillations such as delta waves^94^ and K-complexes^95^ also decreases with age. Nonetheless, it is unclear why these age-related changes in SWS exist. One explanation is the loss of healthy neurons due to aging, which were formerly responsible for complex synchronized brain activities during SWS.^96^ Prospective aging studies have also identified the prefrontal cortex, where most slow oscillations originate from, to experience the greatest loss in grey matter volume as compared to other brain regions.^97,98^ Recently, alterations in the anterior thalamic radiation (i.e. major white matter fiber bundle connecting the prefrontal cortex and thalamus), were found to be associated with poor sleep-wake cycles in older adults ‘at-risk’ of dementia.^99^ This raises the possibility that white matter alterations could disrupt sleep neuro-circuitry and thus the propagation of delta waves. Therefore, the role of white matter tract disruption in nocturnal autonomic functions during SWS warrants future investigation.

The findings of the present investigation should be considered with the following caveats. Our sample, while extremely well characterized with respect to clinical, sleep and HRV features, did not have investigation for Alzheimer’s disease biomarkers, such as amyloid and/or tau positron emission tomography scanning, so we were unable to delineate the likely pathology underpinning their cognitive profile. Given a lack of consensus on sympathetic indices of HRV,^100^ examination of the interaction between sympathetic activities during sleep and functional connectivity in the brain was not possible for the current study. Future studies should explore new ways to address this research gap. There is regular contact between our patients and the radiographer between MRI sequences, thereby ensuring consciousness at the onset of fMRI acquisition, and the sequence is only 6.5 min long. Nevertheless, it remains a consideration that some of the participants may have fallen asleep during parts of their scan or undergone microsleeps. Unfortunately, this limitation is inherent to this type of study, where simultaneous EEG-fMRI is not available. Furthermore, while the brainstem has been a crucial area of the CAN that has attracted previous research interest, acquisition of functional data with an acceptable signal-to-noise ratio from the brainstem is challenging, due to the proximity to cerebrospinal fluid and major arteries, which confound the functional connectivity blood-oxygen-level-dependent signal with physiological noise.^101^ In addition, the brainstem nuclei are small and difficult to segment or define.^102^ These issues may be overcome by using higher-field MR scanners where reliable acquisition of functional data at much higher resolutions is possible.^34^ Lastly, despite simultaneous acquisition of fMRI and HRV often being employed for the purpose of physiological artefact removal,^103^ measuring HRV during sleep with simultaneous fMRI would allow a closer examination of the brain–heart coupling and further elucidate crucial CNS-ANS associations.

In conclusion, our study offers new innovations in our understanding of brain–heart relations by demonstrating that HF-HRV during SWS is linked to the strength of brain coupling across core and broader brain regions of the CAN in older adults ‘at risk’ of dementia. Given these relationships were not found for WASO or REM sleep HF-HRV, it is likely that parasympathetic regulation during NREM sleep, particularly SWS, and resting-state brain network cohesiveness might be intrinsically linked or could be a manifestation of underlying evolving neurodegenerative pathology. Due to the cross-sectional nature of the study, it would be crucial to determine the directionality of the two processes using a longitudinal approach in the future to better understand the brain–heart modulation of the ANS, and to determine how perturbations of the system have prognostic utility for ongoing cognitive decline. This may allow for the early identification of autonomic dysfunction that may be etiologically linked to disease trajectory. In turn, such advances could in turn inform the formulation of targeted interventions for either the cardiovascular system or sleep systems, with the aim to optimize cognition or ideally, to slow or prevent the cognitive decline leading to dementia.

## Abbreviations

ANS =: autonomic nervous system
BMI =: body mass index
CAN =: central autonomic network
CIRS-G =: cumulative illness rating scale geriatric version
CNS =: central nervous system
ECG =: electrocardiogram
fMRI =: functional magnetic resonance imaging
GDS =: geriatric depression scale-short form
HRV =: heart rate variability
HF-HRV =: high-frequency heart rate variability
MMSE =: mini-mental state examination
MNI =: Montreal Neurological Institute
MRI =: magnetic resonance imaging
NREM =: non-rapid eye movement
REM =: rapid eye movement
rsfMRI =: resting-state functional magnetic resonance imaging
SWS =: slow wave sleep
